# Isoflurane anesthesia and sleep deprivation trigger delayed and selective sleep alterations

**DOI:** 10.1038/s41598-024-64975-9

**Published:** 2024-06-18

**Authors:** Leesa Joyce, Clara Carrillo Mas, Veronica Meedt, Matthias Kreuzer, Gerhard Schneider, Thomas Fenzl

**Affiliations:** Department of Anesthesiology & Intensive Care, School of Medicine, Technical University of Munich, Ismaninger Strasse 22, 81675 Munich, Germany

**Keywords:** Neuroscience, Circadian rhythms and sleep, Cognitive neuroscience, Neural circuits

## Abstract

Isoflurane anesthesia (IA) partially compensates NREM sleep (NREMS) and not REM sleep (REMS) requirement, eliciting post-anesthetic REMS rebound. Sleep deprivation triggers compensatory NREMS rebounds and REMS rebounds during recovery sleep as a result of the body’s homeostatic mechanisms. A combination of sleep deprivation and isoflurane anesthesia is common in clinical settings, especially prior to surgeries. This study investigates the effects of pre-anesthetic sleep deprivation on post-anesthetic sleep–wake architecture. The effects of isoflurane exposure (90 min) alone were compared with the effects of isoflurane exposure preceded by experimental sleep deprivation (6 h, gentle handling) on recovery sleep in adult mice by studying the architecture of post-anesthetic sleep for 3 consecutive post-anesthetic days. Effects of isoflurane anesthesia on recovery sleep developed only during the first dark period after anesthesia, the active phase in mice. During this time, mice irrespective of preceding sleep pressure, showed NREMS and REMS rebound and decreased wakefulness during recovery sleep. Additionally, sleep deprivation prior to isoflurane treatment caused a persistent reduction of theta power during post-anesthetic REMS at least for 3 post-anesthetic days. We showed that isoflurane causes NREMS rebound during recovery sleep which suggests that isoflurane may not fully compensate for natural NREMS. The study also reveals that isoflurane exposure preceded by sleep deprivation caused a persistent disruption of REMS quality. We suggest that preoperative sleep deprivation may impair postoperative recovery through lasting disruption in sleep quality.

## Introduction

Sleep is a fundamental physiological process needed for maintaining overall health and wellbeing, along with other processes^1^. Sustaining a good quality sleep is especially critical when recovering from illness and medical procedures^2–4^. Nevertheless, post-anesthetic/post-surgical sleep disturbances are common, potentially slowing the recovery process^5^. One of the most commonly applied volatile anesthetic drugs has been isoflurane^6^, which causes numerous post-anesthetic short term sleep disturbances^7,8^. However, inconsistency persists on its specific effects. In humans, isoflurane exposure is known to cause a shift from deeper to lighter non-rapid eye movement sleep (NREMS), but has no effects on rapid eye movement sleep (REMS)^9^. Some studies reported mechanistic similarities between isoflurane-induced slow wave activity (SWA) and natural sleep-induced SWA^10–13^ whereas others suggest that these two states are different while the former may partially compensate for the latter^14–16^. In rats it was shown that the physiological status during isoflurane exposure is similar to natural sleep and post-anesthetic effects are independent of dosage^17^. Same findings were reported from studies in rabbits showing that NREMS-like processes are present during isoflurane exposure, although NREMS was reduced during post-anesthetic sleep causing sleep disturbances without affecting REMS^18^. In contrast, studies in mice suggested a homeostatic REMS load after isoflurane exposure^19^. At present, isoflurane is thought to build a homeostatic load only for REMS, inducing a post-anesthetic REMS rebound. Such a homeostatic load is also induced through sleep deprivation, resulting in a NREMS rebound^20^ and REMS rebound^14,21,22^. In the clinical environment it is common for patients to experience pre-operative sleep impairments^23–26^, similar to sleep deprivation. Recent studies suggested that preoperative sleep deprivation is associated with post-operative cognitive decline in humans^27,28^ and rodents^29^ and increased pain sensitivity after surgery^23,30^.

Both, sleep deprivation and isoflurane anesthesia elicit homeostatic response, disrupting the regular sleep–wake architecture. Interestingly an interaction between pre-anesthetic sleep deprivation and anesthesia seems to result in different effects on post-anesthetic sleep compared to those derived from anesthesia or sleep deprivation alone^13,14,31^. However, this interaction is not well understood. The present study investigates the effects of the combination of sleep deprivation and isoflurane anesthesia on post-anesthetic sleep by comparing the trajectory of sleep recovery for three subsequent days after isoflurane anesthesia preceded by sleep deprivation to that of the three subsequent days after isoflurane anesthesia alone.

## Results

### Comparison of sleep architecture between Baseline1 and Baseline2

**Figure 1:**
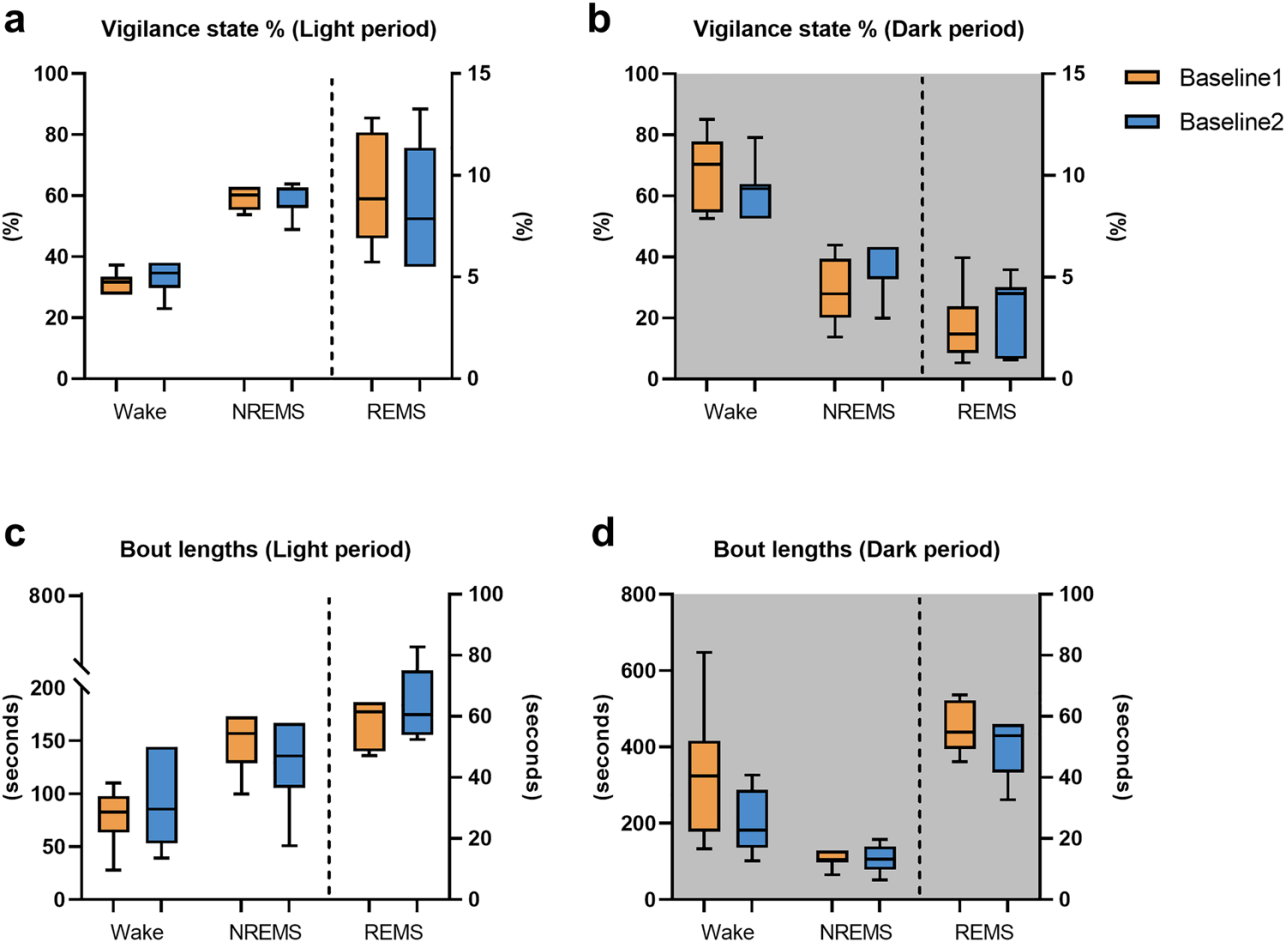
Comparison of temporal characteristics of sleep and wakefulness between Baseline1 (orange) and Baseline2 (blue). Vigilance state percentages (**a,b**) and bout lengths (**c,d**) for Wake, NREMS and REMS are compared between Baseline1 and Baseline2 during light and dark periods separately using Wilcoxon signed rank test (*p* < 0.05), n = 7. The data are represented with the median, the 1st quartile and the 3rd quartile. Paired comparisons for each mouse are represented using gray connecting lines. White background: lights ON/inactive period, gray background: lights OFF/active period.

Vigilance state percentages during light period (Fig. 1a) and dark period (Fig. 1b) were compared between Baseline1 and Baseline2. The two baselines did not show significant differences in the total percentage of Wake, NREMS and REMS though 5 out of 7 mice showed a decreasing trend from Baseline1 to Baseline2 in the percentages of REMS during the light period (Fig. 1a) and in the percentages of wakefulness (Wake) during the dark period (Fig. 1b). Bout lengths of Wake, NREMS and REMS during the light period (Fig. 1c) and the dark period (Fig. 1d) were compared between Baseline1 and Baseline2. No statistically significant differences in the bout lengths of the two baselines were found. However, in 5 out of 7 mice, Baseline2 showed a decrease in the bout lengths of NREMS during the light period (Fig. 1c), as compared to Baseline1.

**Figure 2:**
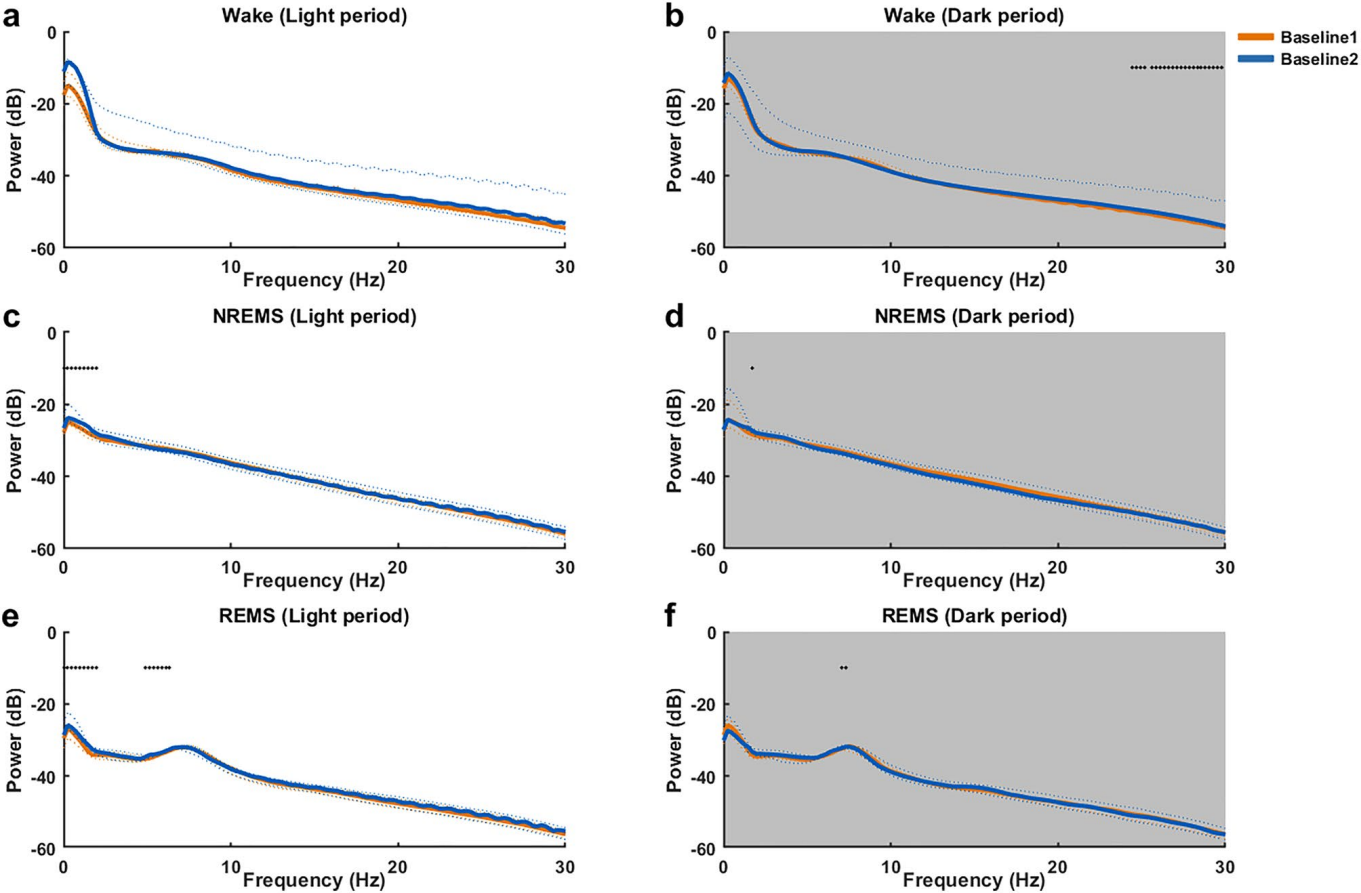
Comparison of spectral characteristics of sleep and wakefulness between Baseline1 (orange) and Baseline2 (blue). PSDs (decibel (dB)) for Wake (**a,b**), NREMS (**c,d**), and REMS (**e,f**) for the two baselines are separately compared at each frequency bin (range 0.5–30 Hz; resolution: 0.25 Hz) during both light and dark periods. The solid lines represent the median and the dotted lines represent the 1st and the 3rd quartile. *p < 0.05 (Wilcoxon signed rank test), n = 7, white background: lights ON / inactive period, gray background: lights OFF/active period.

Power spectral densities (PSDs) for Wake, NREMS and REMS were compared between Baseline1 and Baseline2 for light and dark periods separately. PSDs of Baseline1 and Baseline2 during Wake in the light period (Fig. 2a) did not have significant differences. During Wake in the dark period (Fig. 2b), Baseline 2 showed significantly higher power for frequencies between 24.4 and 30 Hz compared to Baseline1. During NREMS in the light period (Fig. 2c), Baseline 2 showed higher power in the frequency range of 0.1–2.2 Hz compared to Baseline1. PSDs of Baseline1 and Baseline2 during NREMS in the dark period (Fig. 2d) did not have significant differences. During REMS in the light period (Fig. 2e), Baseline 2 showed higher power in the frequency range of 0.1–2.2 Hz and 4.8–6.6 Hz compared to Baseline1. PSDs of Baseline1 and Baseline2 during REMS in the dark period (Fig. 2f) did not have significant differences. To account for such differences between the baselines, all the parameters of post- isoflurane anesthesia (P-IA) and post-sleep deprivation/isoflurane anesthesia (P-SD/IA) for further analysis were calculated as percentage changes from Baseline1 and Baseline2, respectively.

### Recovery of sleep architecture during inactive periods

**Figure 3:**
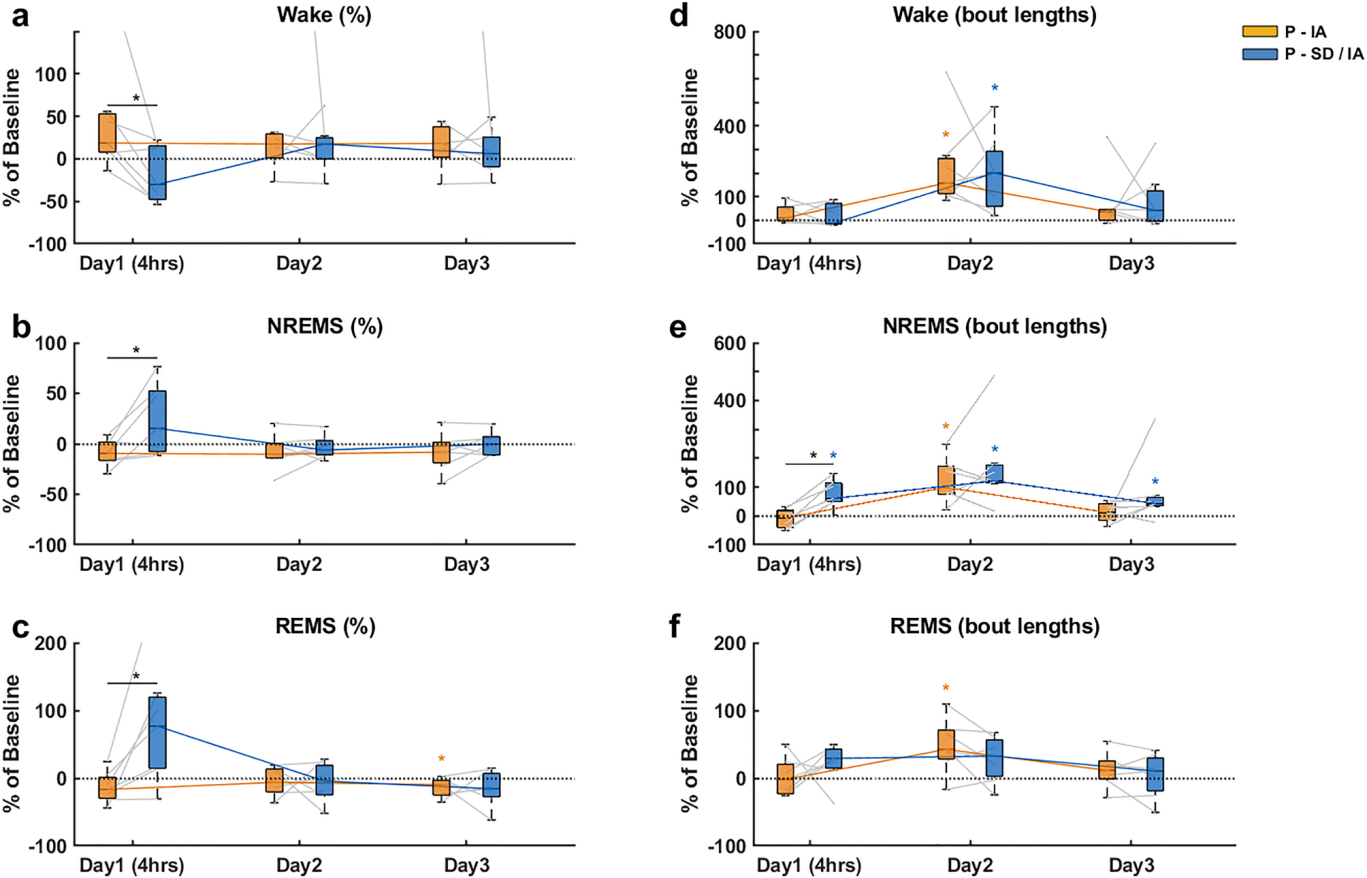
Comparison of temporal characteristics of sleep and wakefulness during the light periods on the post-experimental days. Vigilance state percentages (Wake (**a**), NREMS (**b**), REMS (**c**)) and bout lengths (Wake (**d**), NREMS (**e**), REMS (**f**)) for 3 consecutive days of P-IA and P-SD/IA are shown as percentage change from Baseline1 and Baseline2 respectively. The black dotted line at 0 represents the baselines. The data are represented with the median, the 1st quartile and the 3rd quartile. Paired comparisons for each mouse are represented using gray connecting lines. Trajectory of sleep recovery through the consecutive days of P-IA and P-SD/IA are indicated as color coded slopes. An asterisk over a line segment represents a significant difference between P-IA and P-SD/IA. The color-coded asterisks represent a significant difference from the baseline. *p < 0.05 (Wilcoxon signed rank test), n = 7, white background: lights ON/inactive period.

For day 1, 2 and 3, relative vigilance state percentages and bout lengths of P-IA were compared against that of P-SD/IA. Wake percentages (Fig. 3a) of P-SD/IA on day1 was significantly lower (*p = 0.0313, r = 0.831) than that of P-IA. During P-IA on day1, 6 out of 7 mice showed an increasing trend in Wake percentages from Baseline1. The recovery slopes of Wake percentages between P-IA and P-SD/IA across 3 days did not differ significantly from each other. NREMS percentages (Fig. 3b) of P-SD/IA on day1 was significantly higher (*p = 0.0313, r = 0.831) than that of P-IA. During P-IA on day1, 6 out of 7 mice showed a decreasing trend in NREMS percentages from Baseline1. The recovery slopes of NREMS percentages between P-IA and P-SD/IA across 3 days did not differ significantly from each other. REMS percentages (Fig. 3c) of P-SD/IA on day1 was significantly higher (*p = 0.0156, r = 0.894) than that of P-IA. During P-IA on day1, 5 out of 7 mice showed a decreasing trend in REMS percentages from Baseline1. Conversely, 6 out of 7 mice showed an increasing trend in REMS percentages during P-SD/IA on day1 as compared to Baseline2. REMS percentages of P-IA on day3 was significantly lower (*p = 0.0313, r = 0.831) than Baseline1. The recovery slopes of REMS percentages of P-SD/IA from day1 to day2 was negative and steeper (*p = 0.0312, r = 0.831) than that of P-IA.

Wake bout lengths (Fig. 3d) of P-IA and P-SD/IA on day2 showed a significant increase (*p = 0.0156, r = 0.894) from their respective baselines. Wake bout lengths of both P-IA and P-SD/IA across 3 days had similar recovery slopes. NREMS bout lengths (Fig. 3e) of P-IA on day2 showed a significant increase (*p = 0.0156, r = 0.894) from Baseline1.

NREMS bout lengths of P-SD/IA on day1, day2 and day3 were significantly higher (*p = 0.0156, r = 0.894; *p = 0.0156, r = 0.894; and *p = 0.0312, r = 0.831 respectively) than Baseline2. NREMS bout lengths of P-SD/IA on day1 were significantly higher (*p = 0.0313, r = 0.831) than P-IA. NREMS bout lengths of both P-IA and P-SD/IA across 3 days had similar recovery slopes. REMS bout lengths (Fig. 3f) of 6 out of 7 mice showed an increasing trend during P-SD/IA on day1 as compared to Baseline2. REMS bout lengths of P-IA on day2 were significantly higher (*p = 0.0312, r = 0.831) than Baseline1. The recovery slopes of REMS bout lengths of P-IA from day1 to day2 were positive and steeper (*p = 0.0312, r = 0.831) than that of P-SD/IA.

**Figure 4:**
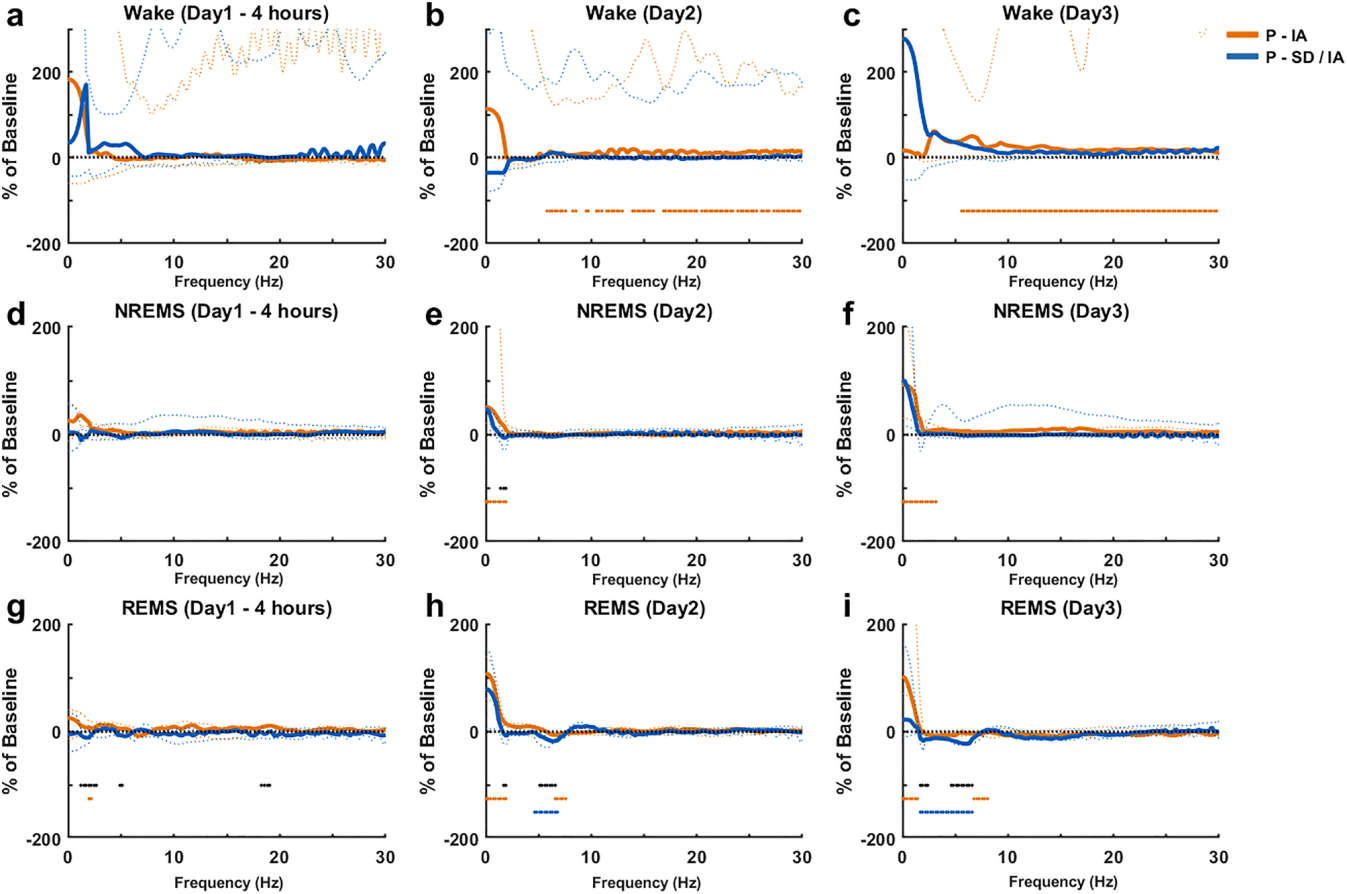
Comparison of spectral characteristics of sleep and wakefulness during the light periods on the post-experimental days. PSDs of Wake (Day1 (**a**), Day2 (**b**), Day3 (**c**)), NREMS (Day1 (**d**), Day2 (**e**), Day3 (**f**)) and REMS (Day1 (**g**), Day2 (**h**), Day3 (**i**)) of P-IA and P-SD/IA are shown as percentage change from Baseline1 and Baseline2 respectively. The black dotted line at 0 represents the baselines. The solid lines represent the median and the dotted lines represent the 1st and the 3rd quartile. The black asterisks represent a significant difference between P-IA and P-SD/IA. The color-coded asterisks represent a significant difference from the baseline. *p < 0.05 (Wilcoxon signed rank test), n = 7, white background: lights ON/inactive period.

For day 1, 2 and 3, relative PSDs of Wake, NREMS and REMS during P-IA were compared against that of P-SD/IA. Wake-PSDs of P-IA and P-SD/IA on day1 (Fig. 4a) did not differ from each other as well as from the respective baselines.

Wake-PSDs of P-IA on day2 (Fig. 4b) and day3 (Fig. 4c) showed a significant increase in the broad band (5.6–30 Hz) power as compared to Baseline1. NREMS PSDs of P-IA and P-SD/IA on day1 (Fig. 4d) did not differ from each other as well as from the respective baselines. NREMS PSDs of P-IA on day2 (Fig. 4e) showed a significant increase in the frequency range of 0–2.2 Hz as compared to Baseline1 and 1.5–2.2 Hz as compared to P-SD/IA. NREMS PSDs of P-IA on day3 (Fig. 4f) showed a significant increase in the frequency range of 0–3.4 Hz as compared to Baseline1. REMS PSDs of P-IA and P-SD/IA on day1 (Fig. 4g) did not differ from the respective baselines but P-SD/IA showed a significant decrease in the frequency range of 1.2–2.7 Hz and 18.3–19 Hz from that of P-IA. REMS PSDs of P-IA on day2 (Fig. 4h) showed a significant increase in the frequency range of 0–2.2 Hz and a significant decrease in the frequency range of 6.6–7.6 Hz as compared to Baseline1. REMS PSDs of P-SD/IA on day2 showed a significant decrease in the frequency range of 4.6—6.8 Hz and 5.1–6.8 Hz from Baseline2 and P-IA respectively. REMS PSDs of P-IA on day3 (Fig. 4i) showed a significant increase in the frequency range of 0–1.5 Hz and a significant decrease in the frequency range of 6.8—8 Hz, as compared to Baseline1. REMS PSDs of P-SD/IA on day3 showed a significant decrease in the frequency range of 1.7–6.6 Hz from Baseline2 and a significant decrease in the frequency range of 1.7–2.4 Hz and 4.6–6.6 Hz from P-IA.

### Recovery of sleep architecture during active periods

**Figure 5:**
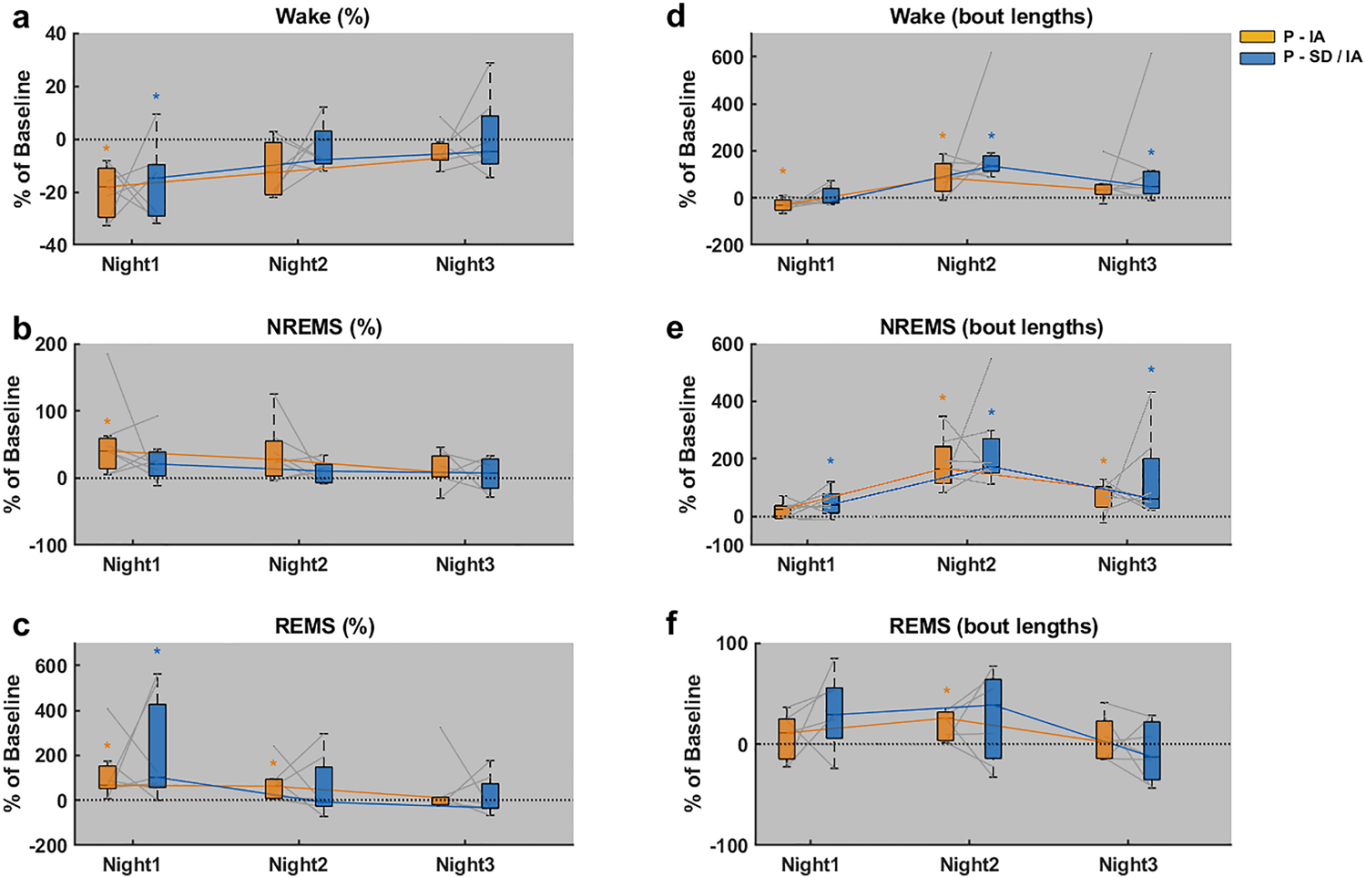
Comparison of temporal characteristics of sleep and wakefulness during the dark periods on the post-experimental days. Vigilance state percentages (Wake (**a**), NREMS (**b**), REMS (**c**)) and bout lengths (Wake (**d**), NREMS (**e**), REMS (**f**)) for 3 consecutive nights of P-IA and P-SD/IA are shown as percentage change from Baseline1 and Baseline2 respectively. The black dotted line at 0 represents the baselines. The data are represented with the median, the 1st quartile and the 3rd quartile. Paired comparisons for each mouse are represented using gray connecting lines. Trajectory of sleep recovery through the consecutive nights of P-IA and P-SD/IA are indicated as color coded slopes. The color-coded asterisks represent a significant difference from the baseline. *p < 0.05 (Wilcoxon signed rank test), n = 7, gray background: lights OFF/active period.

For night 1, 2 and 3, relative vigilance state percentages and bout lengths of P-IA were compared against that of P-SD/IA. Wake percentages (Fig. 5a) of P-IA and P-SD/IA on night1 were significantly lower (*p = 0.0156, r = 0.894; and *p = 0.0312, r = 0.831 respectively) than their respective baselines. The recovery slopes of Wake percentages between P-IA and P-SD/IA across 3 nights did not significantly differ from each other. NREMS percentages (Fig. 5b) of P-IA on night1 were significantly higher (*p = 0.0156, r = 0.894) than Baseline1. The recovery slopes of NREMS percentages of P-IA from night2 to night3 were negative and steeper (*p = 0.0468, r = 0.767) as compared to that of P-SD/IA. REMS percentages (Fig. 5c) of P-IA on night1 and night2 were significantly higher (*p = 0.0156, r = 0.894) than Baseline1. REMS percentages of P-SD/IA on night1 were significantly higher (*p = 0.0312, r = 0.831) than Baseline2. The recovery slopes of REMS percentages between P-IA and P-SD/IA across 3 nights did not significantly differ from each other.

Wake bout lengths (Fig. 5d) of P-IA on night1 was significantly lower (*p = 0.0468, r = 0.767) and on night2 was significantly higher (*p = 0.0312, r = 0.831) than Baseline1. Wake bout lengths of P-SD/IA on night2 and night3 were significantly higher (*p = 0.0156, r = 0.894 and *p = 0.0468, r = 0.767 respectively) than Baseline2. Both P-IA and P-SD/IA across 3 nights had similar recovery slopes for Wake bout lengths. NREMS bout lengths (Fig. 5e) of P-IA on night2 and night3 were significantly higher (*p = 0.0156, r = 0.894 and *p = 0.0468, r = 0.767 respectively) than Baseline1. NREMS bout lengths of P-SD/IA on night1, night2 and night3 were significantly higher (*p = 0.0468, r = 0.767; *p = 0.0156, r = 0.894; and *p = 0.0156, r = 0.894 respectively) than Baseline2. Both P-IA and P-SD/IA across 3 nights had similar recovery slopes for NREMS bout lengths. REMS bout lengths (Fig. 5f) of P-IA on night2 was significantly higher (*p = 0.0156, r = 0.894) than Baseline1. The recovery slopes of REMS bout lengths for P-SD/IA from night2 to night3 were negative and steeper (*p = 0.0312, r = 0.831) than that of P-IA.

**Figure 6:**
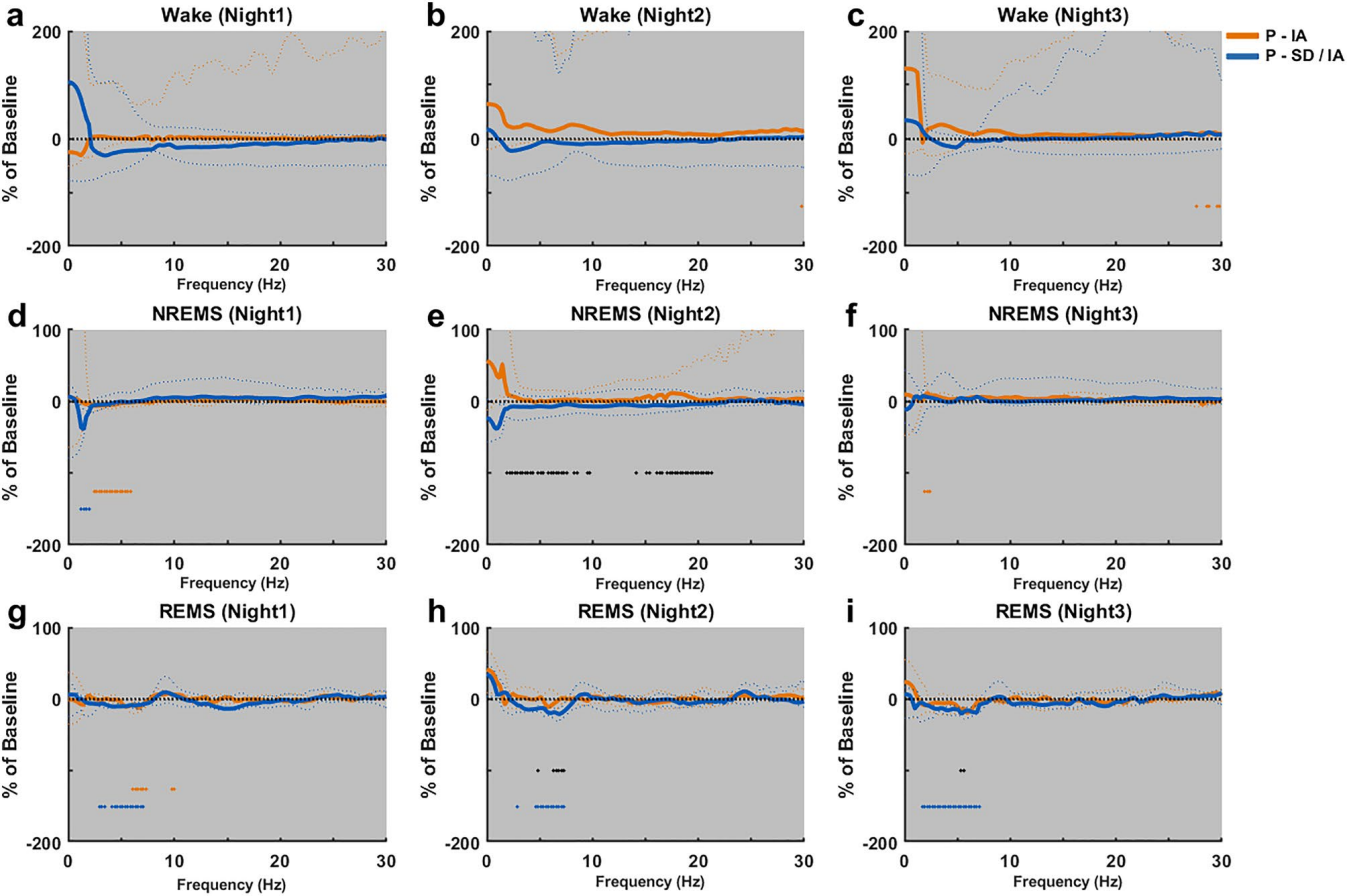
Comparison of spectral characteristics of sleep and wakefulness during the dark periods on the post-experimental days. PSDs of Wake (Night1 (**a**), Night2 (**b**), Night3 (**c**)), NREMS (Night1 (**d**), Night2 (**e**), Night3 (**f**)) and REMS (Night1 (**g**), Night2 (**h**), Night3 (**i**)) of P-IA and P-SD/IA are shown as percentage change from Baseline1 and Baseline2 respectively. The black dotted line at 0 represents the baselines. The solid lines represent the median and the dotted lines represent the 1st and the 3rd quartile. The black asterisks represent a significant difference between P-IA and P-SD/IA. The color-coded asterisks represent a significant difference from the baseline. *p < 0.05 (Wilcoxon signed rank test), n = 7, gray background: lights OFF/active period.

For night 1, 2 and 3, relative PSDs of Wake, NREMS and REMS during P-IA were compared against that of P-SD/IA. Wake PSDs of P-IA and P-SD/IA on night1 (Fig. 6a), night2 (Fig. 6b) and night3 (Fig. 6c) did not show significant differences from each other as well as from the respective baselines. NREMS PSDs of P-IA on night1 (Fig. 6d) had lower power than Baseline1 in the frequency range of 2.4–6.1 Hz. NREMS PSDs of P-SD/IA on night1 had lower power than Baseline2 in the frequency range of 1.2–2.1 Hz. NREMS PSDs of P-IA on night2 (Fig. 6e) were significantly higher than P-SD/IA in the frequency range of 1.9–7.8 Hz and 16.1–21.4 Hz. NREMS PSDs of P-IA on night3 (Fig. 6f) had higher power than Baseline1 in the frequency range of 1.9–2.6 Hz. REMS PSDs of P-IA on night1 (Fig. 6g) had lower power than Baseline1 in the frequency range of 6.1–7.5 Hz. REMS PSDs of P-SD/IA on night1 had lower power than Baseline2 in the frequency range of 2.9–7.3 Hz. REMS PSDs of P-SD/IA on night2 (Fig. 6h) showed a significant decrease in the frequency range of 4.6–7.5 Hz from Baseline2 and a significant decrease in the frequency range of 6.3–7.5 Hz from P-IA. REMS PSDs of P-SD/IA on night3 (Fig. 6i) showed a significant decrease in the frequency range of 1.7–7.3 Hz as compared to Baseline2.

## Discussion

Sleep deprivation in the form of impaired sleep behavior due to stress or anxiety is a common clinical situation prior to an upcoming surgery under general anesthesia. It was recently shown in mice that short-term memory damages in cognitive behavioral tasks were more severe with short sleep plus anesthesia than with short sleep or anesthesia separately, severe long-term memory deficits were observed only after short sleep plus anesthesia^29^. In humans, reduced sleep prior to surgery (with anesthetic exposure) revealed potential postoperative cognitive dysfunctions^27^. The present study investigated the prolonged effects of IA together with sleep deprivation on three consecutive post-anesthetic days in mice. The results clearly revealed lasting effects on post-anesthetic sleep–wake architecture, suggesting impairments in recovery from IA preceded by SD.

In contrast to previous reports^13^, the first 4 post-anesthetic recovery hours of P-IA on day1 showed increased Wake proportions (Fig. 3a), when compared to its baseline. Moreover, when compared to the first 4 h of P-SD/IA on day1, the corresponding P-IA showed higher Wake (Fig. 3a) and lower sleep (Fig. 3b,c) proportions. One explanation for this could be linked to the change in home cage behavior after IA due to reduced animal well-being, induced through the inhalation anesthesia per se^32^. In this context, animal well-being refers to the condition of an animal devoid of stress or discomfort that can be determined by the animal’s innate behaviors, including home-cage activities such as nesting and appropriate sleeping postures. This increased Wake and reduced sleep was not seen after SD/IA. This behavioral alteration of increased Wake likely subsided as a consequence of heightened sleep pressure induced by sleep deprivation. An alternative argument for the absence of NREMS and REMS rebound immediately after IA may be the reduction of sleep pressure due to the substitution of natural sleep by isoflurane-driven effects. This explanation seems only partly true as NREMS and REMS rebound was recorded during the following recovery hours in the dark period (Fig. 5b,c). Though the first post-anesthetic 4 h of P-IA and P-SD/IA were different, the sleep recovery on the subsequent days followed a similar trajectory, eventually reaching baseline levels on day3. The delayed normalization of Wake, NREMS and REMS was accompanied by an increase in bout lengths especially on day2 (Fig. 3d–f respectively). This is in line with previous studies showing that sleep recovery is mainly driven through alterations in bout lengths of the vigilance states^22,33,34^. Furthermore, despite a relatively swift titration of IA compared to prior studies^13,14,19^, the present study reaffirms previously observed effects of SD and IA on post-anesthetic sleep.

In rats and mice, isoflurane seems to substitute for natural NREMS, as no NREMS-rebound is described after isoflurane exposure^17,19^, even though isoflurane influences the general sleep architecture. Six hours of isoflurane exposure increased the number of NREMS bouts and decreased NREMS bout lengths, eventually ending up with the same proportion of NREMS, compared to baseline^19^. Also in humans, isoflurane anesthesia does not drive NREMS rebound, although the drug induces transient alterations in nocturnal sleep by increasing the proportions of stage 2 sleep and decreasing that of stage 3 and stage 4 sleep^9^. The present study revealed that a delayed NREMS rebound occurred in the dark period on the day of IA (Fig. 5b), which progressively declined in the following nights. With the introduction of a homeostatic sleep pressure prior to IA, NREMS rebound was recorded directly after IA in the inactive period (Fig. 3b). In this case, since the immediate NREMS rebound in the light period was accompanied by an increase in the NREMS bout lengths (Fig. 3e) causing a recovery, the rebound did not last until the following dark period (Fig. 5b). Irrespective of sleep pressure, isoflurane exposure seems to trigger NREMS rebound. In discordance with most studies, this may indicate that isoflurane may not globally compensate for natural NREMS.

Within the spectral domain, slow wave activity during slow wave sleep (SWS) at sleep stage N3, the best characterized marker of sleep homeostasis and sleep quality^35,36^, seemed to be substituted during isoflurane anesthesia. This was shown in rats where anesthesia with isoflurane or desflurane, both associated with the occurrence of almost continuous slow waves, reduced the SWS rebound after 4 h of sleep deprivation^13^. Interestingly, the present study with a continuous observation for 3 nights revealed that delayed SWS rebound occurred on day2 (Fig. 4e) and day3 (Fig. 4f). Such delayed rebound effects support the hypothesis that isoflurane may only partly compensate for natural NREMS.

Earlier studies have shown that isoflurane anesthesia does not compensate for the homeostatic need for REMS, thus supporting REMS rebound^19,21,37^. Moreover, isoflurane-induced sleep rebound is known to emerge during the active phase in mice^19^, except when isoflurane exposure is preceded by prolonged selective REMS deprivation resulting in immediate REMS rebound in the inactive period^21^. Interestingly, the present results revealed that increasing sleep pressure preceding isoflurane exposure may cause a small REMS rebound during recovery sleep in the inactive period (Fig. 3c). As expected, significant REMS rebound during the active period was observed during both P-IA and P-SD/IA (Fig. 5c).

Though the introduction of pre-anesthetic sleep deprivation did not alter the temporal features of post-anesthetic sleep recovery significantly, a prolonged impact was observed in the spectral domain. Beginning from the dark period on the day of IA (Fig. 6g), the PSD of REMS during P-SD/IA showed a reduction in theta band power, as compared to baseline as well as P-IA in all inactive periods (Fig. 4h,i) and active periods (Fig. 6h,i). From post-experimental day 1 to day 3 of P-SD/IA, the reduction of theta power seemed persistent, indicating a strong effect. Moreover, the reduction of the spectral power expanded beyond theta to delta range. Studies have shown the role of REMS in memory consolidation^38–41^ and emotional memory processing^42,43^, which is directly linked to theta activity during REMS^44,45^. The reduction of theta power during REMS found in the present study may implicate a negative effect of SD/IA on the quality of REMS, potentially affecting emotional memory processing and memory consolidation. Future studies observing extended longitudinal post-anesthetic days may reveal even long-term impact on brain functionality.

It has been clearly shown that repeated application of isoflurane, even when severe, causes only short-term mild distress and temporary impairments in the well-being of mice^46^. Studies have also shown that well-being is shortly affected by repeated isoflurane anesthesia and is milder in male than in female mice. However, the impact on well-being reached stability no later than 8 days after the final instance of repeated anesthesia. Similar results were shown for the impact of repeated isoflurane anesthesia on spatial and psychomotor performance in mice^47^. Repeated anesthesia neither impaired the behavioral performance of young and aged mice nor prolonged cognitive impairments in aged mice. These findings closely align with the accumulated experience in our laboratory over the years and supported the study’s experimental design, which involved well-spaced repeated anesthesia administrations. Moreover, the baselines (Baseline1 and Baseline2) before the experiments were compared with each other (Figs. 1, 2), not revealing significant differences. However, beyond the tested aspects of sleep–wake architecture, sleep/wake behavior may not have been fully recovered after IA and prior to SD/IA. Thus the homeostatic effects of SD/IA may be interpreted carefully. The results are also reported as the percentage changes from the respective baselines, avoiding the influence of the former experiment over the later while interpreting the results.

In spite of the contributions made by this study, several limitations should be acknowledged. The reported effects may vary with different administration protocols and dosages of isoflurane. Likewise, different durations of sleep deprivation have varying effects on subsequent sleep. The observed effects are specific to isoflurane prompting careful considerations when generalizing these results to the human context, where desflurane or sevoflurane are typically administered. Although the commonly used volatile anesthetic drugs desflurane, sevoflurane and isoflurane are all halogenated ethers, their physiological interaction with pre-operative impaired sleep might be different. Moreover, the mice exhibit polyphasic sleep behavior unlike humans, and the homeostatic responses of mice to treatments like anesthesia and sleep deprivation might not be directly comparable to that of humans. In mice, circadian rhythms play a crucial role in regulating sleep–wake cycles, like in humans. These rhythms orchestrate the temporal organization of physiological processes, including sleep architecture over the 24-h period^48^. Notably, circadian influences govern the propensity for specific sleep stages, such as REMS and “deep” NREMS, exhibiting temporal variations throughout the day^49–53^. Consequently, the timing of experimental interventions relative to the endogenous circadian rhythm of mice emerges as a critical determinant of experimental outcomes. In our experimental design (light phase starting at 9 am), the administration of the second dose of isoflurane (IA), following a period of 6-h sleep deprivation (SD), was scheduled for 3 pm, whereas the first dose of isoflurane (IA) occurred at 9 am. This discrepancy in timing suggests that the comparison of sleep architecture between these two groups may not solely be contingent upon the sleep load accrued during the preceding SD period but may also be influenced by the inherent variations in sleep regulation dictated by the circadian cycle. Thus, interpretations of post-experimental sleep architecture should account for the interplay between sleep debt accumulation and the circadian timing of experimental interventions. This emphasizes the significance of future investigations understanding circadian effects of experimental interventions. Furthermore, the study exclusively included male mice. While acknowledging that the estrous status of female mice may contribute to variations in sleep, it is anticipated that such effects would be relatively modest in comparison to those stemming from genotype differences^54^. Moreover, we opted out experiments on females primarily due to our apprehension regarding the potential uncontrollable olfactory influence on the sleep/wake behavior of male mice in the presence of female animals.

Since the present data were collected as part of a subsequent experiment performed in one cohort of mice, the LFP electrodes implanted in the nuclei of the sleep/wake promoting pathway for later experiments may have interfered with global sleep/wake behavior of the animals. The comparison of basal sleep behavior of mice with LFP implantations versus basal sleep behavior of mice without LFP implantations under identical experimental conditions in our laboratory revealed no significant differences (Supplementary Figs. S1, S2). Our consistent reuse of laboratory animals primarily stems from our commitment to adhere to the principles of the 3R guidelines of laboratory animal welfare, which are systematically applied in all our experiments. Fully aware of the challenges in translational research and the generalizability of data from basic biomedical research, our studies have focused on illuminating fundamental mechanisms underlying the interactions of sleep and anesthesia. With this in mind, clinical research should address identical experimental protocols regarding the combination of sleep deprivation and anesthesia, bridging the gap between fundamental knowledge and clinical practice.

Based on a relatively large effect size, alpha error and medium power together with our long experience in EEG studies with identical and similar group sizes (please refer to Refs.^55–60^), we are confident to identify significant/relevant EEG-based results in small group sizes, as shown in the present study. This approach is supported by the 3R guidelines to use as few animals as possible in our experiments.

The current study shows the occurrence of NREMS rebound in the active period on the day of isoflurane treatment and the occurrence of SWS rebound during the inactive periods on the following days. This indicates that isoflurane might not globally compensate for natural NREMS. Findings from the present study also showed that sleep deprivation prior to isoflurane administration had immediate effects on temporal domains as well as lasting effects on spectral domains of post-anesthetic sleep–wake behavior. Prevention of pre-anesthetic, clinically driven sleep deprivation holds the strong potential to reduce post-operative impairments in sleep homeostasis.

## Methods

### Animals

Seven male C57BL/6N mice (Charles River Laboratories GmbH, Germany) aged between 12 and 18 weeks (BW: 24 g to 27 g) were used in the present study. Epidural electroencephalogram electrodes (EEG) and subcortical local field potential electrodes (LFPs) were implanted on the animals and basal chronic recordings of sleep/wake behavior were acquired for 1 week. As these animals were also part of a subsequent study including recordings from LFPs, all electrodes were implanted in a single surgery (refer to the last paragraph of the discussion and the supplementary information). During the experiments, the mice were housed individually under a 12/12-h light/dark cycle (lights ON/OFF: 9 am/pm, Zeitgeber time ZT, temperature: 22 °C ± 2 °C, humidity: 55% ± 10%) with ad libitum access to food and water. The home cage was enriched with shredded paper for bedding and wooden chewing bars, which were standardized across all experimental conditions and groups. All experimental procedures were approved by the Committee of Animal Health and Care of the State of Upper Bavaria, Germany (ROB-55.2–2532.Vet_02–19–121). Care of laboratory animals and experiments were performed in accordance with the recommendations of the European Union for the care and use of laboratory animals and in accordance with ARRIVE guidelines^61^. The study was not preregistered with the Open Science Framework.

### Surgery and electrode implantation

For the electrode implantation surgery, anesthesia was induced in a sealed acrylic glass chamber with 4 vol% isoflurane (CP-Pharma Handelsgesellschaft GmbH, Germany). To assess loss of righting reflex (LORR), the mouse was gently tipped onto its back by tilting the chamber gently. After LORR, the mouse was transferred to a stereotaxic frame (Leica Mikrosysteme Vertrieb GmbH, Germany). Throughout the surgery, anesthesia was maintained at 1.8–2.0 vol% isoflurane with a flow rate of 192 ml/min. Body temperature of 37 °C was maintained using a homeothermic monitoring system (Harvard Apparatus, USA). For analgesia, Carprofen (4 mg/kg BW, Carprofen, Zoetis, Germany) was subcutaneously injected before surgery and Lidocaine Hydrochloride (2%, bela-pharm GmbH & Co. KG, Germany) was applied on the incisions during the surgery. After shaving the head, skin incisions were made to expose the upper cranium and then the periosteum was removed. Three epidural EEG electrodes (left frontal; AP: + 1.78, ML: − 1.17, left parietal; AP: − 2.70, ML: − 1.57, ground electrode; AP: + 0.62, ML: − 3.02), one electromyogram (EMG) electrode (nuchal muscle) and LFPs at the ventrolateral preoptic nucleus (VLPO; AP: + 0.01, ML: − 0.65, DV: − 5.75), the ventral posteromedial nucleus (VPM; AP: − 1.79, ML: − 1.35, DV: − 3.65) and the locus coeruleus (LC; AP: − 5.41, ML: − 0.82, DV: − 3.78) were implanted on the mice. A printed circuit board socket (Preci-Dip, series 861, Delémont, Switzerland) holding all electrodes was attached to the cranium using dental cement (Paladur, Kulzer GmbH, Germany) and two jeweler’s screws. The EEG and the EMG electrodes were made of 24 K gold wires (⌀ 150 µm, Haefner & Krullmann GmbH, Germany). The LFPs were made of stainless-steel wire with perfluoro alkoxy alkane insulation (bare wire ⌀ 76 µm, coated wire ⌀ 140 µm, Science Products GmbH, Germany). Postoperative analgesia (0.067 mg/ml, Carprofen) was administered for 3 days through drinking water.

### Experimental design

**Figure 7:**
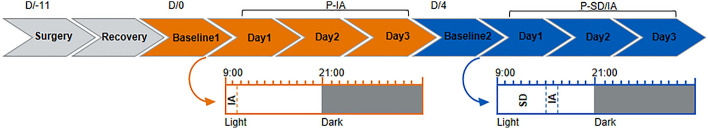
Experimental timeline. The colored blocks represent the subsequent days in the experimental timeline. Baseline1 (D/0) and Baseline2 (D/4) represent the 2 baseline recording days. P-IA Day1 represents the day of experimental anesthesia which was followed by two post-anesthetic EEG recording days (P-IA Day2 and P-IA Day3). P-SD/IA Day1 represents the day of sleep deprivation (SD) and experimental anesthesia (IA) which was followed by two post-anesthetic EEG recording days (P-SD/IA Day2 and P-SD/IA Day3).

10 days after the surgery, baseline EEG (D/0: Baseline1, Fig. 7) was recorded starting at ZT 9 am (Lights ON). Data were acquired for 23 h (12 h light period and 11 h dark period) on each recording day while the last hour of the day was used for animal care and technical maintenance. After Baseline1, isoflurane anesthesia was administered at the beginning of the light period (Day1, Fig. 7). EEG was continuously recorded for the post-anesthetic hours of Day1 (PP-IA Day1), as well as the following 2 days (P-IA Day2 and P-IA Day3) where the mice were allowed to freely behave in the home cage. On day 4, the sequel experiment was started with a second baseline (D/4: Baseline2, Fig. 7). After recording Baseline2, on the following day at the beginning of the light period (Day1, Fig. 7), mice underwent sleep deprivation which lasted for 6 h^20,55,62^. SD was performed in the home cage by gentle handling which involved the manipulation of the bedding and introduction of novel cage enrichments which were strictly consistent across mice^63,64^. SD was immediately followed by IA. EEG was continuously recorded for the remaining hours of Day1 (P-SD/IA Day1), as well as the following 2 days (P-SD/IA Day2 and P- SD/IA Day3) where the mice were allowed to freely behave in the home cage. The present study with its subsequent experiments on the same animal was designed especially in accordance with the guiding principles of the 3Rs (Replacement, Reduction, Refinement: https://www.nc3rs.org.uk/who-we-are/3rs) for the ethical use of animals in scientific research^65^.

### Experimental anesthesia

For the experimental anesthesia, mice were transferred to a transparent acrylic glass chamber (0.006 m^3^) with a hermetic seal and a heating pad. The chamber had a gas inlet and a vent along with a gastight inlet for the recording cable and the gas probe (CAPNOMAC ultima, Datex Ohmeda, Louisville, United States, air volume flow rate: 1.5 l/min, inspiratory oxygen concentration: 0.5). Along with the start of EEG data acquisition, anesthetic administration in the chamber was initiated with 0.2% isoflurane, progressively increased by steps of 0.2% every 2 min until the online observation of the first second of a burst-suppression episode, which served as the marker for a common anesthetic end point for all the mice. Burst suppression is a neurophysiological phenomenon characterized by the alternation between periods of intense electrical activity (bursts) and periods of relative electrical silence (suppression) in the EEG signal^66^. This was immediately followed by a decrease of 0.2% isoflurane concentration every 2 min until 0% isoflurane which led to the termination of the experiment. The cumulative duration of isoflurane exposure, commencing from the initiation of 0.2% isoflurane administration until the onset of burst suppression, followed by a subsequent return to 0% isoflurane concentration within the enclosed glass chamber, spanned approximately 90 min. Of the 90 min, each mouse remained in the state of anesthetic-induced LORR for a minimum duration of 30 min, extending up to a maximum of 35 min.

### Data acquisition

Each mouse was connected to a tethered recording system including headstage/recording cable (1× amplification, custom made, npi electronics GmbH, Germany) along with a commutator (model SL-20, Dragonfly R&D Inc., USA), mounted on a weight-neutral swivel system (custom made, Streicher M., Innsbruck, Austria) facilitating the unrestricted movement of the animal^67^. The EEG and EMG signals were independently amplified (1000×) and band-pass filtered for frequencies between 0.1 and 100 Hz along with a notch filter at 50 Hz (DPA-2FL-Differential Amplifier, npi electronic, Germany). All recording channels were sampled at 250 Hz (Power1401-3A, Cambridge Electronic Design Ltd., UK) and imported to MATLAB—R2019a (MathWorks, USA) for data processing and analyses.

### Data analyses

23 h of EEG and EMG data from 7 recording days (Baseline1, P-IA Day1, P-IA Day2, P-IA Day3, Baseline2, P-SD/IA Day1, P-SD/IA Day2, P-SD/IA Day3) from all 7 mice were divided into two halves namely light period (first 12 h) and dark period (subsequent 11 h) using custom scripts. From the light periods of P-IA Day1 and P-SD/IA Day1, only the 4 h immediately after the experiments were selected for further analysis. EEG- and EMG signals were down-sampled to 125 Hz for sleep scoring. A LABVIEW-based (National Instruments, Austin, TX, USA), semi-automated sleep scoring software^55,68,69^ was used to annotate the vigilance states Wake, NREMS and, REMS to each epoch (4-s-long non-overlapping EEG episodes). The semi-automated sleep scores were manually reviewed by an experienced scorer to ensure the accuracy of vigilance state assignments especially at all vigilance state transitions. Scorers remained blinded to information regarding the experiments and the condition of the mice throughout the assessment process. To compare the recovery sleep across 3 days, bout lengths and vigilance state percentages were calculated as percentage change from respective baselines for each phase (light/dark) separately. Slopes between consecutive days were calculated to compare the trajectory of sleep recovery for both the experiments.

Using the MATLAB inbuilt function pwelch, the PSD of individual vigilance states were calculated separately for light and dark periods for frequencies from 0 to 30 Hz with a frequency resolution of 0.25 Hz for each EEG epoch. PSDs were calculated as percentage change from the respective baselines for each frequency bin for each vigilance state separately.

### Statistical analysis

All statistics were computed using custom scripts in MATLAB. All tests were two-tailed where significance was achieved at a 95% confidence interval (α = 0.05). For comparisons between dependent groups, the Wilcoxon signed rank test was employed and for comparisons between two independent groups (refer to the supplementary information), the Mann Whitney U test was employed. For PSDs, statistical comparisons between groups were done separately for each frequency. All data represented in the graphs show median, the 1st quartile and the 3rd quartile. Box and whisker plots were constructed utilizing the interquartile range (IQR) method, wherein the box represents the range from the lower quartile to the upper quartile. Beyond the upper quartile, a length equivalent to 1.5 times the IQR was determined, and a whisker was extended to the largest observed data point within this range. Similarly, below the lower quartile, a distance of 1.5 times the IQR was measured, and a whisker was drawn to the lowest observed data point within this interval. Any data points beyond the boundaries of the whiskers are depicted as outliers and can be recognized for each figure individually. Absolute *p*-values are listed only for the significant differences (*p* < 0.05). In the PSDs, random significant differences spanning less than 3 consecutive frequency bins were considered irrelevant. For a list of all p-values (significant and non-significant) refer to Supplementary Table S1.

Effect size (r) was calculated as Z statistic divided by the square root of the sample size. The Z statistic was calculated through the signrank function in MATLAB as a test statistic for Wilcoxon signed rank test. The Wilcoxon effect size (r value) ranges from 0 to (almost) 1. An effect size that is greater than or equal to 0.5 represents a large effect.

### Supplementary Information

Supplementary Information.

## Data Availability

The dataset generated and analyzed during the current study are not publicly available due to ongoing research using the same set of data but will be available from the corresponding author on reasonable request.
