# Supplementary material for: Isoflurane anesthesia and sleep deprivation trigger delayed and selective sleep alterations
Source: Sci Rep. 2024 Jun 18;14:14060. doi: 10.1038/s41598-024-64975-9 (PMC11189473; doi:10.1038/s41598-024-64975-9)
Supplement: Supplementary file 1 — Supplementary Information. [file 41598_2024_64975_MOESM1_ESM.pdf]

Supplementary Information

A control study examined whether the LFP electrodes, implanted into sleep promoting (VLPO), wake promoting (LC) and thalamic (VPM) areas interfered with the natural sleep/wake behavior of the mice. For this, 6 mice matching strain, age, gender and body weight from different experiments in our laboratory were randomly selected (EEG-electrodes with the identical design, no LFP electrodes). In identical experimental conditions, 23 hours of baselines from this particular group of 6 mice (without LFPs) were directly compared with Baseline1 of the 7 mice (with LFPs) from the present experimental group. Temporal (Supplementary Fig. S1) and spectral (Supplementary Fig. S2) parameters of sleep/wake behavior between mice with and without LFPs did not reveal any significant differences.

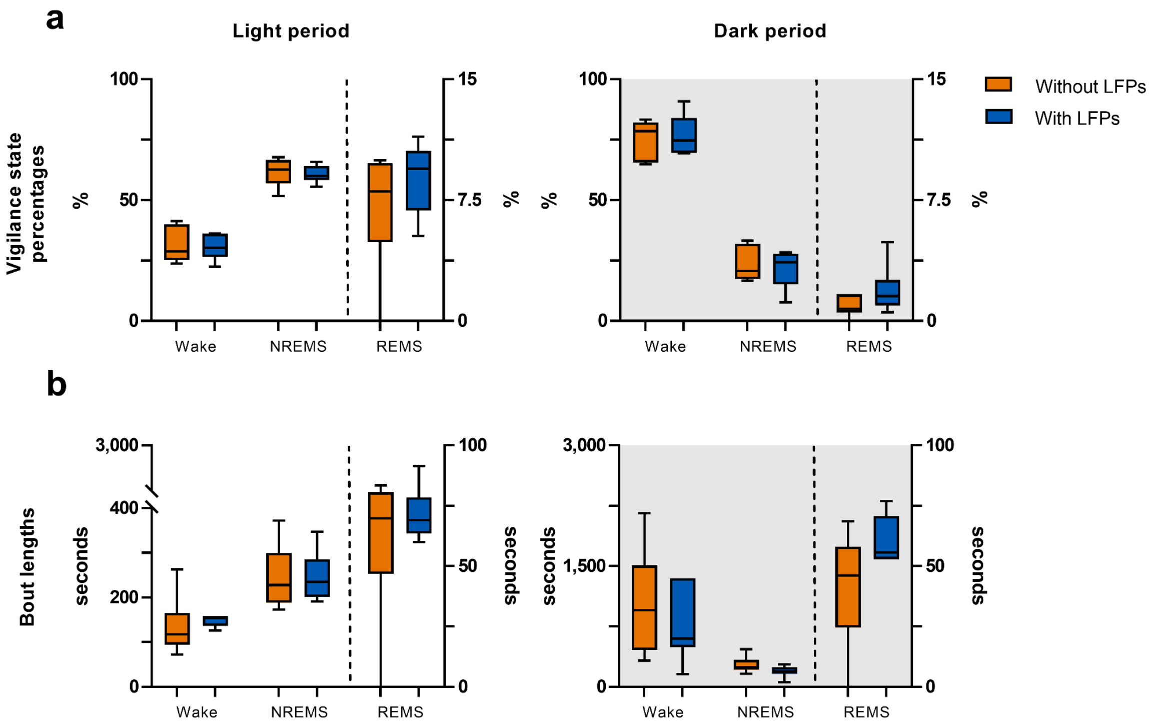

Supplementary Figure S1. Comparison of temporal characteristics of sleep and wakefulness between mice with LFPs (n=7, blue) and without LFPs (n=6, orange). No significant differences could be detected. For the 2 groups, for both light and dark periods (shown in white and gray backgrounds respectively), (a) vigilance state percentages (%), and (b) bout lengths (seconds) were separately compared for Wake, NREMS and REMS. REMS data are plotted on the right y-axis. The data are shown as the median, the 1st quartile and the 3rd quartile. *$p < 0.05$ (two tailed Mann Whitney U test).

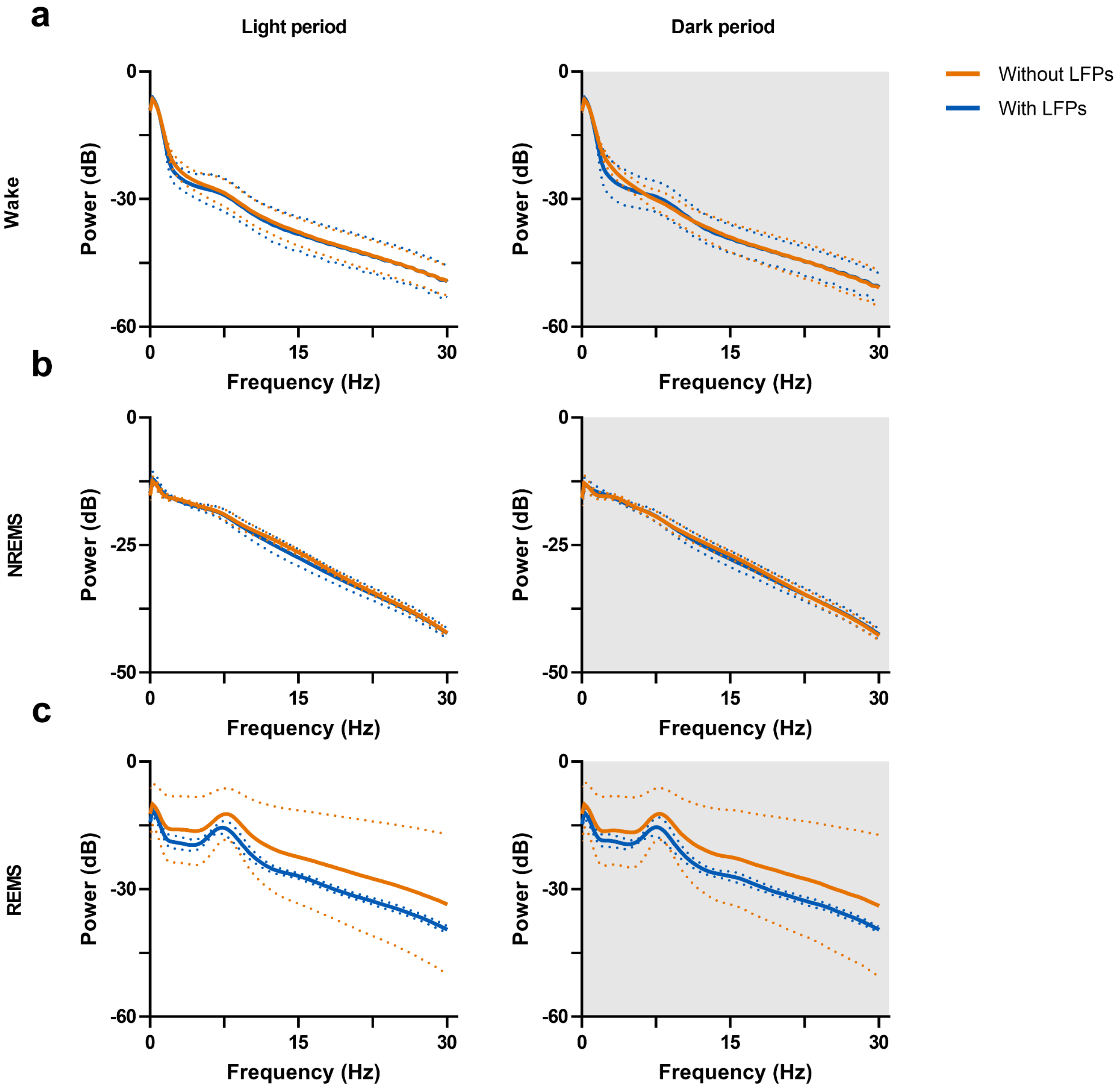

Supplementary Figure S2. Comparison of spectral characteristics of sleep and wakefulness between mice with LFPs (n=7, blue) and without LFPs (n=6, orange). No significant differences could be detected. For the 2 groups, for both light and dark periods (shown in white and gray backgrounds respectively), PSDs for (a) Wake, (b) NREMS, and (c) REMS are separately compared at each frequency bin (range: 0 - 30 Hz; resolution: 0.25 Hz). The PSDs in decibels (dB; logarithmic) are shown as the median (solid line), the 1st and the 3rd quartile (dotted lines), *$p < 0.05$ (two tailed Mann Whitney U test).

**Figure 1: Baseline1 vs Baseline2**									
	**Wake**	**NREMS**	**REMS**						
**2.a**	0,4688	0,375	0,375						
**2.b**	0,2969	0,2969	0,4688						
**2.c**	0,5781	0,375	0,6875						
**2.d**	0,375	0,9375	0,8125						
**Figure 3: Light period**									
	**Day1 (4hrs)**			**Day2**			**Day3**		
	P - IA vs Baseline1	P - SD/IA vs Baseline2	P - IA vs P - SD/IA	P - IA vs Baseline1	P - SD/IA vs Baseline2	P - IA vs P - SD/IA	P - IA vs Baseline1	P - SD/IA vs Baseline2	P - IA vs P - SD/IA
**4.a**	0,0781	0,21875	**0,0313**	0,21875	0,375	0,2969	0,21875	0,46875	0,4688
**4.b**	0,15625	0,21875	**0,0313**	0,375	0,375	0,296875	0,375	0,8125	0,296875
**4.c**	0,109375	0,109375	**0,0156**	0,6875	0,46875	0,6875	**0,03125**	0,109375	1
**4.d**	0,296875	0,6875	0,6875	**0,015625**	**0,015625**	0,8125	0,078125	0,15625	1
**4.e**	0,578125	**0,015625**	**0,0131**	**0,015625**	**0,015625**	0,4688	0,578125	**0,03125**	0,2188
**4.f**	0,8125	0,15625	0,375	**0,03125**	0,078125	0,2969	0,375	0,8125	0,4688
**Figure 3: Recovery slopes**									
	**Day 1-2**	**Day 2-3**							
**4.a**	0,375	0,21875							
**4.b**	0,078125	0,21875							
**4.c**	**0,03125**	0,9375							
**4.d**	0,6875	0,296875							
**4.e**	0,578125	0,375							
**4.f**	**0,03125**	0,6875							
**Figure 5: Dark period**									
	**Day1 (4hrs)**			**Day2**			**Day3**		
	P - IA vs Baseline1	P - SD/IA vs Baseline2	P - IA vs P - SD/IA	P - IA vs Baseline1	P - SD/IA vs Baseline2	P - IA vs P - SD/IA	P - IA vs Baseline1	P - SD/IA vs Baseline2	P - IA vs P - SD/IA
**6.a**	**0,015625**	**0,03125**	0,9375	0,078125	0,46875	0,2188	0,21875	0,6875	0,5781
**6.b**	**0,015625**	0,078125	0,578125	0,078125	0,21875	0,21875	0,15625	0,9375	0,6875
**6.c**	**0,015625**	**0,03125**	0,9375	**0,015625**	0,8125	0,2188	0,9375	0,296875	0,5781
**6.d**	**0,046875**	0,8125	0,375	**0,03125**	**0,015625**	0,375	0,109375	**0,046875**	1
**6.e**	0,21875	**0,046875**	0,375	**0,015625**	**0,015625**	0,8125	**0,046875**	**0,015625**	0,8125
**6.f**	0,46875	0,078125	0,21875	**0,015625**	0,15625	0,46875	0,8125	0,578125	0,375
**Figure 5: Recovery slopes**									
	**Day 1-2**	**Day 2-3**							
**6.a**	0,15625	0,296875							
**6.b**	0,375	**0,046875**							
**6.c**	0,21875	0,578125							
**6.d**	0,296875	0,296875							
**6.e**	0,6875	0,8125							
**6.f**	0,109375	**0,03125**							
**Supplementary Figure S1:**									
	**Light period**			**Dark period**					
	Wake	NREMS	REMS	Wake	NREMS	REMS			
**Suppl.1a**	0,8357	0,6282	0,4452	0,7308	0,6282	0,1684			
**Suppl.1b**	0,0734	0,6282	>0.9999	0,8357	0,2343	0,1807			

Supplementary Table S1. Summarized *p*-values for Fig. 1, 3, 5 and Supplementary Fig. S1.
